# Design and Testing of a Seedling Pick-Up Device for a Facility Tomato Automatic Transplanting Machine

**DOI:** 10.3390/s24206700

**Published:** 2024-10-18

**Authors:** Zhicheng Liu, Lu Shi, Zhiyuan Liu, Jianfei Xing, Can Hu, Xufeng Wang, Long Wang

**Affiliations:** 1 College of Mechanical and Electrical Engineering, Tarim University, Alar 843300, China; Liuzc1026@outlook.com (Z.L.); 120219022@taru.edu.cn (L.S.); liuz030914@outlook.com (Z.L.); 120200012@taru.edu.cn (J.X.); 120140004@taru.edu.cn (C.H.); wxf@taru.edu.cn (X.W.); 2 Modern Agricultural Engineering Key Laboratory at Universities of Education Department of Xinjiang Uygur Autonomous Region, Tarim University, Alar 843300, China; 3 Xinjiang Production and Construction Corps (XPCC) Key Laboratory of Utilization and Equipment of Special Agricultural and Forestry Products in Southern Xinjiang, Alar 843300, China

**Keywords:** agriculture, transplanting, tomato, seedling device, yolov5

## Abstract

At present, tomato transplanting in facility agriculture is mainly manual operation. In an attempt to resolve the problems of high labor intensity and low efficiency of manual operation, this paper designs a clip stem automatic transplanting and seedling picking device based on the yolov5 algorithm. First of all, through the study of the characteristics of tomato seedlings of different seedling ages, the age of tomato seedlings suitable for transplanting was obtained. Secondly, the improved yolov5 algorithm was used to determine the position and shape of tomato seedlings. By adding a lightweight upsampling operator (CARAFE) and an improved loss function, the feature extraction ability and detection speed of tomato seedling stems were improved. The accuracy of the improved yolov5 algorithm reached 92.6%, and mAP_0.5 reached 95.4%. Finally, the seedling verification test was carried out with tomato seedlings of about 40 days old. The test results show that the damage rate of the device is 7.2%, and the success rate is not less than 90.3%. This study can provide a reference for research into automatic transplanting machines.

## 1. Introduction

Facility agriculture denotes the mechanization and intellectualization of agricultural production. With the rapid development of China’s economy and the improvement of science and technology, facility agriculture has gradually become more popular [1,2]. The development of facility agriculture requires the support of related technical equipment and machinery. At present, the demand for facility machinery in China is increasing each year [3,4]. As of 2022, the area covered by facility-based agriculture in China reached 2.85 million hectares, making China the country with the largest area covered by facility-based agriculture [5]. Currently, the southern Xinjiang region is vigorously developing facility agriculture, and the transplanting of facility tomatoes is mainly manual. Manual operations are not only costly but also inefficient due to repeated operations. Therefore, to improve the efficiency of transplanting operations and reduce the cost of operation, it is necessary to research automatic transplanting and seedling-picking devices.

Much research has been performed on crop transplanting devices. Ren et al. [6] designed a retractable tomato seedling-picking device that was then manufactured according to the simulation parameters optimized by the ADAM algorithm. The device was shown to achieve a good transplanting effect. Zhou et al. [7] investigated a seedling picking device of a pepper plug seedling transplanter and proposed an ‘eagle-mouth’ seedling pick-up trajectory. The experimental results revealed the feasibility of the approach. Lai et al. [8] designed a directional transplanting device for Panax notoginseng roots based on the yolov5s algorithm, with the optimal working parameters obtained through experiments. Although the directional qualification rate reached 89.87%, the device requires further optimization. Han et al. [9] developed a door-frame swing seedling pick-up device. The optimal operating parameters and working conditions were obtained through performance testing, and the quality of seedling pick-up was confirmed. Hu et al. [10] designed a flexible clamping seedling extraction mechanism using the bionic principle for seedling injury during the transplantation of chili pepper seedlings. The authors derived the stress range of the clamp from finite element analysis, and the seedling extraction success and injury rates were determined as 94.14% and 3.28%, respectively, indicating a good transplantation effect. Khadatkar et al. [11] designed an automatic transplanting machine to ensure efficient transplanting operations. The final transplanting success rate of the automatic transplanting machine was determined as 90.3% by analyzing and testing the manipulator, but the machine was expensive.

With the recent continuous development of deep learning, deep learning algorithms have been widely used in crop picking and object grasping [12,13]. Chen et al. [14] proposed a yolov3-based tea bud recognition and picking point extractability approach to improve the completeness of tea bud picking, yet the recognition accuracy of the algorithm requires strengthening. Sun et al. [15] proposed a yolo-GG algorithm to assist robots in recognizing grasping, thus improving the grasping accuracy. Zhang et al. [16] proposed a fast detection and grasping method, based on the improved Faster R-CNN, which can effectively achieve detection and fetching. Wang et al. [17] proposed an improved yolov5n model for cherry tomato automatic picking machinery that can realize target recognition during cherry tomato mechanical picking and subsequently achieve mechanical automatic picking. Yan et al. [18] proposed a Mask R-CNN Positioning of Picking Point for Tea Shoots (MR3P-TS) model to reduce the cost of manual tea picking. The authors proved the effectiveness of the model in recognizing overlapping buds and its strong generalization and robustness ability. Mariam et al. [19] designed an automatic picking system for jasmine harvesting, using yolov5 to detect and locate the flowers for gripping. Although the results revealed the strong detection ability of the system, it can still be further optimized.

In summary, the tomato hole tray seedling transplanting process is predominantly a manual procedure. Thus, this paper adopts machine vision to design a tomato hole tray seedling transplanting device for seedling picking based on stem clamping. We integrate the lightweight upsampling operator Content-Aware ReAssembly of Features (CARAFE) and improve the loss function to enhance the grasping accuracy of the tomato burrowing seedling stems. The coordinates of the tomato stems are then obtained by the depth camera and combined with a mechanical claw to complete the grasping of the tomato hole tray seedlings.

## 2. Materials and Methods

### 2.1. Morphological Analysis of Tomato Hole Tray Seedlings

**Figure 1:**
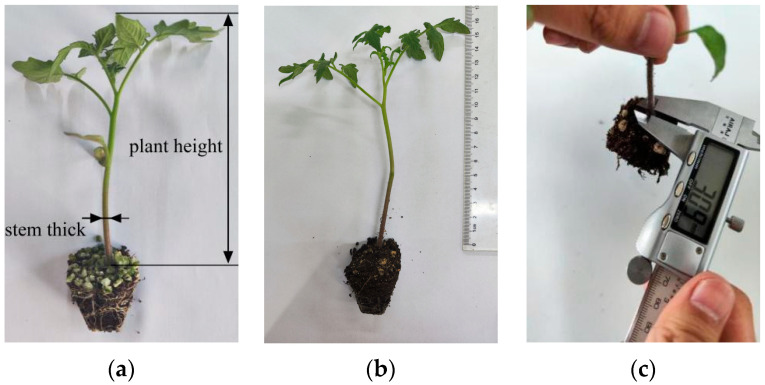
Measurement procedure of the tomato seedling morphological characteristics. (**a**) Morphological characteristics of tomato seedlings; (**b**) plant height measurements; (**c**) stem thickness measurements.

To understand the physiological characteristics and morphological changes in tomato seedlings at different growth stages and to determine the appropriate transplanting period, this paper selected tomato plants at 30, 35, 40, 45, and 50 days of age. The selected tomato hole-pot seedlings had the same substrate ratios and were irrigated an equal number of times with the same amount of water. We consider the stem thickness as the average diameter of the stem located in the range of 10 to 40 mm above the surface of the substrate, and plant height as the distance from the surface of the substrate to the top of the seedling [20,21]. The stem thickness and plant height of the tomato seedling hole tray seedlings were measured by digital vernier calipers and a straightedge, respectively. A total of 30 tomato hole tray seedlings were taken at each seedling age for the determination of the morphological parameters. Figure 1 presents the experimental process.

The morphological parameters of the tomato hole tray seedlings were measured at five seedling ages and analyzed to determine the optimum transplanting period. Table 1 reports the measured morphological parameters.

The average plant height and stem thickness growth of the tomato seedlings varied more from 30 to 40 days, and less from 40 to 50 days. This indicates that the seedling age exhibited less growth variation after 40 days. Therefore, tomato hole tray seedlings with an age of about 40 days or more are suitable for transplanting.

### 2.2. Device Design and Operating Principle

#### 2.2.1. Device Design

**Figure 2:**
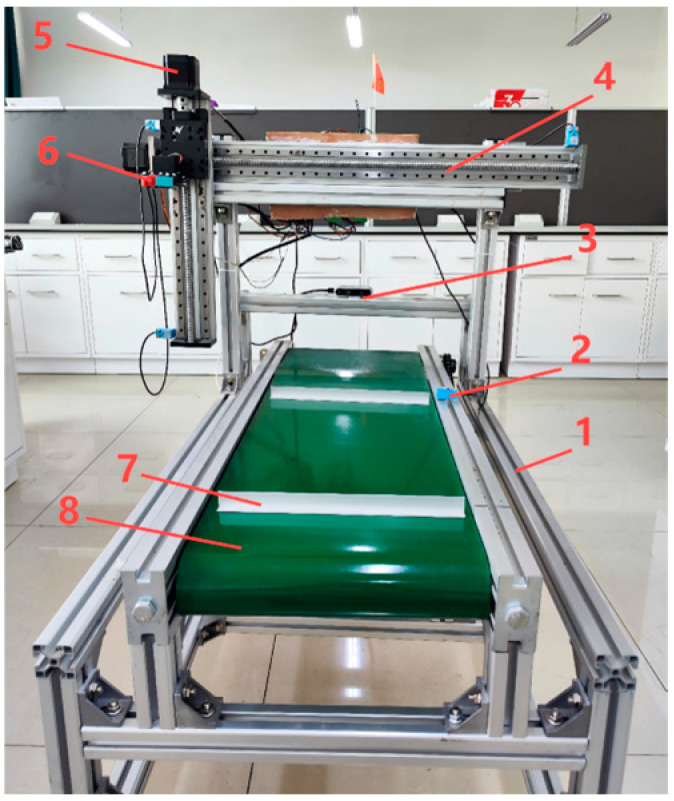
Device physical diagram. 1. Frame. 2. Limit switch. 3. Camera. 4. Ball screw. 5. Stepping motor. 6. Flexible clamping jaws. 7. Cavity plate fixed stopper. 8. Conveyor belt.

The seedling extraction device is the core of the transplanting machine. At present, seedling extraction devices are mainly based on stem clamping, ejecting, air blowing, etc. [22]. The device in this paper combines the yellow sand substrate planting mode in southern Xinjiang with the manual seedling picking working mode for a clamping stem-type tomato hole tray seedling transplanting and picking device. The device employs camera depth data to obtain the tomato hole tray seedling location and hand–eye calibration to control the mechanical claw, thus completing the seedling-picking operation. Figure 2 depicts the proposed device.

#### 2.2.2. Operation Principle of the Proposed Device

The working process of the seedling pick-up device is as follows: (1) The plug seedlings are placed on the conveyor belt and the plug surface is limited by the baffle fixed on the conveyor belt. (2) The camera, fixed behind the rack, identifies and locates the plug seedlings. (3) The ball screw drives the mechanical claw horizontally and vertically to the corresponding position. When the conveyor belt starts to move and triggers the limit switch, it stops, and the mechanical claw grabs the tomato seedling stem. (4) The mechanical claw closes along the longitudinal ball screw back to the starting point and then along the transverse ball screw back to the origin; at the same time, the conveyor belt moves back by one hole distance, and the distance of one hole is 38 mm. The seedling-picking process then terminates.

### 2.3. Tomato Seedling Stem Identification

#### 2.3.1. Tomato Seedling Stem Recognition Analysis

**Figure 3:**
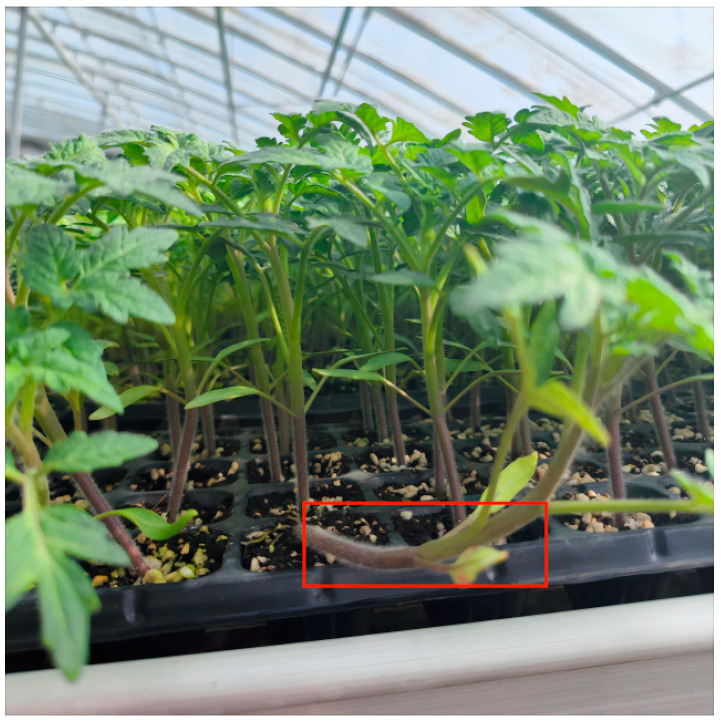
The stem is seriously distorted.

In the process of transplanting and picking up tomato plug seedlings, due to the different growth conditions of tomato seedlings, the appearance of their stems is different. The stems of tomato seedlings with good growth conditions should be straight or slightly bent, and there will inevitably be some seedlings with poor growth conditions in the process of seedling raising. These seedlings with poor growth conditions will affect the transplanting survival rate of tomato planting after being transplanted, so the seedlings with poor growth conditions should not be taken in the process of seedling picking. The appearance characteristics of the seedlings with poor growth conditions are characterized by severe stem bending, as shown in Figure 3.

**Figure 4:**
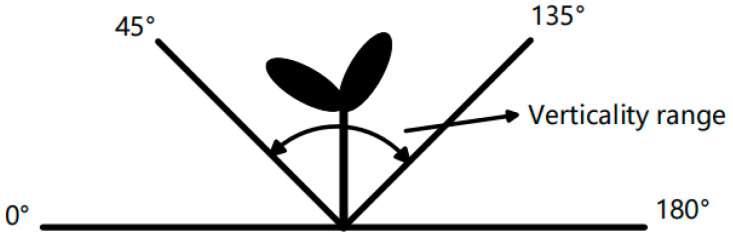
Verticality range.

The bending degree of the stem of tomato seedlings is called uprightness. Verticality refers to the angle β between the main stem of tomato seedlings and the horizontal direction of the plug. The evaluation index of the uprightness of the potted seedling is 45° < β < 135°. When β is in the range of this angle, the tomato seedlings can be transplanted [23]. The range of stem uprightness is shown in Figure 4.

#### 2.3.2. Calculation of Tomato Seedling Stem Coordinates

**Figure 5:**
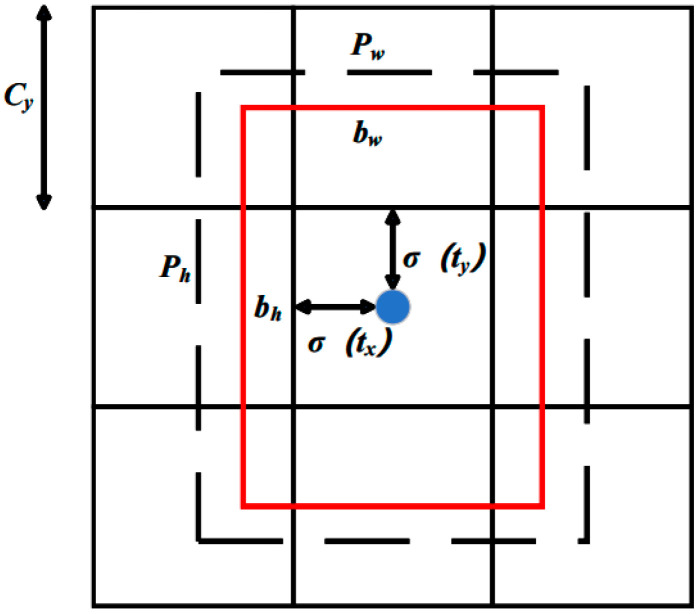
Detection of the object position in the image.

The depth camera is key for the recognition and grasping processes as it provides scene information for the device and connects the three-dimensional real world with the two-dimensional graphic plane [24]. The target detector is output as an RGB image by the camera and passed through the yolov5 network for target detection. When the camera detects the tomato seedling stem, it detects and extracts its position in the image (Figure 4). In Figure 5, C_x_ and C_y_ are the coordinates of the upper-left corner of the center point region; P_w_ and P_y_ are the width and height of the anchor point, respectively, σ(t_x_) and σ(t_y_) are the distance between the center point of the prediction box and the upper-left corner, respectively; and σ is the logistic regression function.

If the center coordinates of the detected object are in a grid, it can be used to detect the object. The formula for calculating the actual position and size of the detected object is described as follows:

(1) $$\left\{\begin{array}{c} b_{x}=2\sigma \left(t_{x}\right)-0.5+C_{x}\\ b_{y}=2\sigma \left(t_{y}\right)-0.5+C_{y}\\ b_{w}=P_{w}\times {\left[2\sigma \left(t_{w}\right)\right]}^{2}\\ b_{h}=P_{h}\times {\left[2\sigma \left(t_{h}\right)\right]}^{2} \end{array}\right.,$$

where t_w_ and t_h_ denote the predicted width and height offsets, respectively. According to b_x_, b_y_, b_w_, and b_h_, the coordinates of the detected object in pixel coordinates can be obtained. The camera internal reference obtained from the calibration experiment is then transformed to finally obtain the coordinates of the detected object in the camera coordinate system.

## 3. Tomato Seedling Stem Detection Model Based on yolov5

### 3.1. yolov5 Target Detection Network

**Figure 6:**
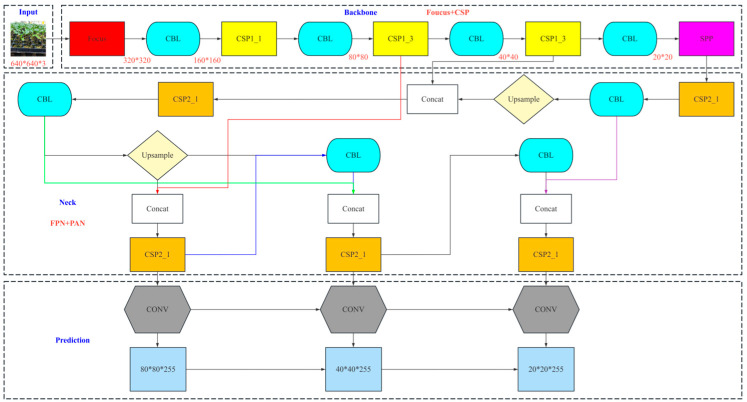
Network structure diagram of yolov5.

yolov5 is a target detection algorithm based on deep learning, which has the characteristics of high efficiency and flexibility. The network structure of yolov5 is mainly composed of an input, backbone, neck, and prediction. The network structure of yolov5 is shown in Figure 6. According to the size of the model and the amount of calculation, yolov5 can be divided into yolov5s, yolov5m, yolov5l, and yolov5x. We employ yolov5s in this paper.

**Figure 7:**
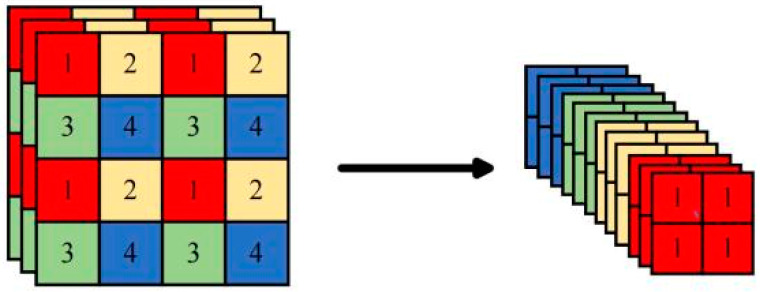
Slicing operation of the FOCUS model in yolov5.

The network structure of yolov5 includes the backbone, neck, and head. The task of the backbone is to perform feature extraction on the input image and gradually enhance the representation of the feature map using the CSPDarknet53 architecture. The CSPDarknet53 consists of the FOCUS module, the CBL module, and the SPP module. The FOCUS module is based on the slicing operation (Figure 7). The CBL module is used for feature extraction and consists of three operations, CONV, BN, and LeakyRelu, which improve the feature extraction. To ensure the fixed size of the input feature map, we employ the SPP module.

Based on the backbone, a set of simple and effective feature pyramid modules are added in the neck. These modules can adaptively focus on features in different regions, thus improving the quality and accuracy of feature representations. The head is composed of three convolution layers and is used to output the results of the target detection, including the object category, border position, and confidence [25,26]. Its working principle can be divided into three steps: feature extraction, target detection, and post-processing. First, yolov5 uses the backbone network to extract the features of the input image at different scales and generates a high-dimensional feature map to represent the input image. To better adapt to object detection tasks of different scales, yolov5 also uses a feature pyramid module to add multiple branches, each with different receptive field sizes, such that target features of different scales can be captured. yolov5 then uses the predicted head to further process the feature map to output the target detection results. The prediction head is composed of three convolution layers, and the last layer outputs the detection results (category, confidence, and border position). The category and confidence predictions are separated from the border position prediction, which improves the interpretability and adaptability of the model. Finally, yolov5 uses post-processing techniques, such as non-maximum suppression (NMS) and confidence thresholding, to remove redundant borders in the detection results and outputs the final target detection results [27]. In this way, yolov5 can accurately identify and locate objects in the input image.

In summary, the backbone network of yolov5 employs convolution and pooling operations for the feature extraction of input images of different sizes. After the backbone network, a feature pyramid module is added, where each branch has a different receptive field size to adapt to different scale object detection tasks [28]. Finally, yolov5 uses the predicted head to output the target detection results. In this process, a three-layer convolution is used for feature extraction and synthesis.

### 3.2. Establishment of the Data Sets

We collected 1400 photographs of tomato plug seedlings that had reached the transplanting period in a greenhouse seedling shed. Poor-quality photographs were removed from the data set.

**Figure 8:**
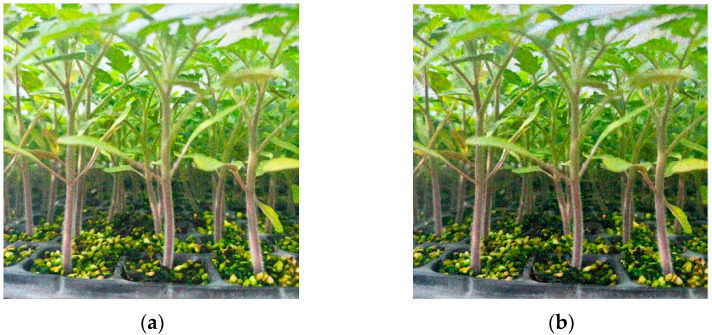

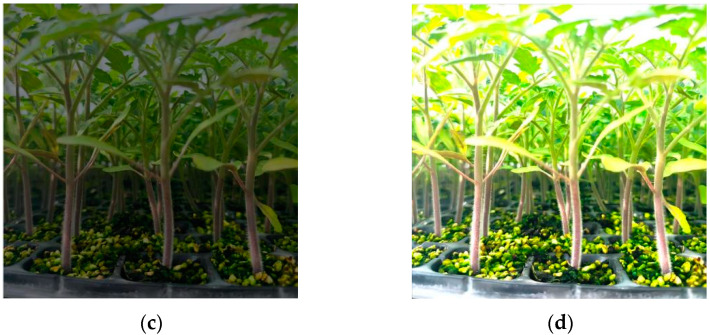
Example image of tomato plug seedlings after data processing. (**a**) Gaussian noise; (**b**) salt and pepper noise; (**c**) darkness; (**d**) brightness.

To better identify the stem of the tomato seedlings and improve the stem recognition accuracy, we use added noise and brightness transformation for data processing. Compared with other types of noise, Gaussian noise, and salt and pepper noise are simpler. They can simulate the noise in the actual scene. Gaussian noise can simulate the random noise in the actual scene, and salt and pepper noise can simulate the sudden noise in the actual scene, to improve the robustness of the model. Therefore, this paper chooses Gaussian noise and salt and pepper noise when adding noise. The brightness transformation is to change the color value of each channel of the pixel. Darkness and brightness are used in this paper. The data enhancement increases the training data diversity, such that the tomato seedling stem detection model can better adapt to the object recognition task under varying scenarios and conditions. Through a series of processing steps of the original image, more images are generated, allowing the model to learn richer features and context information. Figure 8 presents an example image after data processing.

After data enhancement processing, the number of tomato plug seedlings pictures was 2400, and 2400 tomato plug seedlings pictures were divided into the training set, test set, and verification set in proportions of 80%, 10%, and 10%, respectively.

Before detection, the tomato seedling images were labeled in the captured images. The image labeling includes a rectangular box, with the tomato seedling stem in the middle of the box to reduce the interference caused by the excess background. The position of the frame is determined by the two diagonal coordinates.

### 3.3. Experimental Environment and Experimental Methods

This experiment is implemented on Pycharm2022, using Python3.9.7 as the programming language. In all experiments, we set the batch size to 16, the initial learning rate to 0.01, the number of iterations to 200, and the image size to 640 × 640.

### 3.4. Improvement of the yolov5 Algorithm

In this paper, we improve the original yolov5 algorithm to enhance the accuracy of tomato seedling stem recognition and reduce the leakage detection rate. In particular, we add the lightweight upsampling operator CARAFE and improve the loss function to enhance the algorithm’s detection effect on tomato seedling stems.

#### 3.4.1. Addition of CARAFE

**Figure 9:**
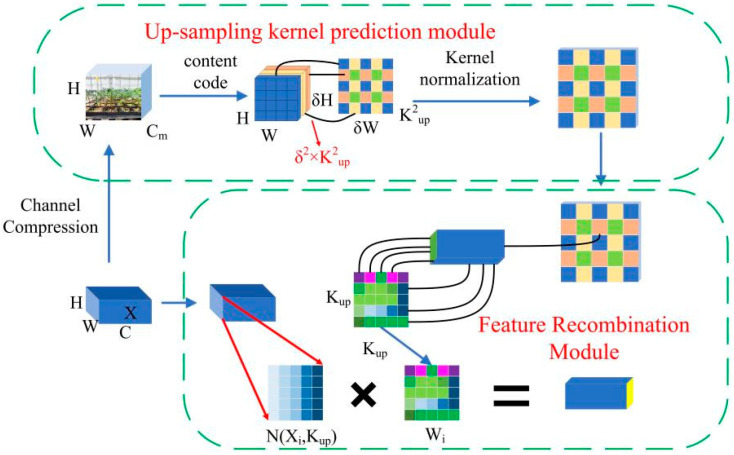
CARAFE framework diagram.

In yolov5, bilinear interpolation is usually used for upsampling, and the upsampling perception field of this method is very small [29]. Therefore, to further improve the performance of yolov5, this paper adds a lightweight upsampling operator (CARAFE) to the yolov5 model. It consists of two main modules: the upsampling kernel prediction module and the feature recombination module. Figure 9 presents the framework diagram for CARAFE. The upsampling kernel prediction module predicts a learnable recombination kernel based on the content of each target position, thereby achieving adaptive convolution operations. The feature recombination module is to use the predicted recombination check to recombine the features to obtain more discriminative features [30,31].

CARAFE has a larger perceptual horizon compared to bilinear interpolated upsampling, allowing it to aggregate contextual information over a large reception domain. Moreover, the content perception of CARAFE varies with the sample, namely it supports instance-specific content-aware processing and can dynamically generate adaptive kernels. The FPN (feature pyramid network) architecture of CARAFE enhances the upsampling and significantly outperforms the original FPN framework in terms of performance. The addition of CARAFE to the network can effectively improve the performance of the model and keep it lightweight due to its lightweight characteristics.

#### 3.4.2. Improvement of the Loss Function

In the improved yolov5 proposed in this paper, we replace the original loss function CIoU (Complete-IoU) with EIoU (Efficient-IoU). CIoU is a measure of the distance between two bounding boxes and is an improvement over the traditional IoU (intersection over union) algorithm. CIoU not only calculates the IoU but also adds a correction factor, which enables it to better adapt to objects with different aspect ratios and proportions [32]. CIoU is determined as follows:

(2) $$L_{\mathrm{CIoU}}=1-\mathrm{IoU}+\frac{\rho^{2}\left(b,b^{\mathrm{gt}}\right)}{c^{2}}+\alpha V,$$

where

(3) $$V=\frac{4}{\pi^{2}}{(\arctan\frac{w^{2}}{h^{\mathrm{gt}}}-\arctan\frac{w}{h})}^{2},$$

(4) $$\alpha =\left\{\begin{array}{l} 0\;\;\;\;\;\;\;\;\;\;\;\;\;\;\;\;\;\;\;\;\;\;,\mathrm{if}\;\mathrm{IoU}\;<\;0.5\\ \frac{V}{\left(1\;-\mathrm{IoU}\right)+V}\;\;\;\;\;\;\;,\mathrm{if}\;\mathrm{IoU}\;\geq \;0.5 \end{array}\right.,$$

where V is the normalization of the length–width ratio difference between the prediction box and the true box, b and b^gt^ are the center points of the two rectangular boxes (the prediction box and the true box), respectively, c is the diagonal distance of the closure area of the two rectangular boxes, α is the weight coefficient, ρ is the Euclidean distance between b and b^gt^, and w, h and w^gt^, h^gt^ are the height and width of the prediction box and the height and width of the true box, respectively.

Although CIoU has improved compared to DIoU, there are still shortcomings. CIoU cannot reflect the real difference between width and height and their confidence, which sometimes hinders the effective optimization similarity of the model. Moreover, with CIoU, when either w and h increase, the other value must be reduced, and, thus, both variables cannot simultaneously increase or decrease. Therefore, scholars proposed EIoU, which further splits and optimizes the aspect ratio in CIoU and includes focal focusing technology to optimize the quality of the anchor frame [33]. EIoU is described as follows:

(5) $$L_{\mathrm{EIoU}}=L_{\mathrm{IoU}}+L_{\mathrm{dis}}+L_{\mathrm{asp}}=1\;-\mathrm{IoU}+\frac{\rho^{2}\left(b,b^{\mathrm{gt}}\right)}{c^{2}}+\frac{\rho^{2}\left(w,w^{\mathrm{gt}}\right)\;}{C_{w}^{2}}+\frac{\rho^{2}(h,h^{\mathrm{gt}})}{C_{h}^{2}},$$

(6) $$L_{\mathrm{EIoU}}=L_{\mathrm{IoU}}+L_{\mathrm{dis}}+L_{\mathrm{asp}}=1\;-\mathrm{IoU}+\frac{\rho^{2}\left(b,b^{\mathrm{gt}}\right)}{c^{2}}+\frac{\rho^{2}\left(w,w^{\mathrm{gt}}\right)}{C_{w}^{2}}+\frac{\rho^{2}(h,h^{\mathrm{gt}})}{C_{h}^{2}},$$

where C_w_ and C_h_ are the width and height of the smallest external frame covering the two boxes, respectively. Equation (6) reveals that the EIoU is composed of three pieces, namely the overlap loss, the center distance loss, and the width–height loss. The original overlap area and centroid distance are maintained in EIoU. Moreover, the width–height loss enhances the convergence speed. Focal loss is also added, which improves the problem of unbalanced training samples and increases the accuracy. EIoU also overcomes the inability of CIoU to simultaneously decrease/increase the width and height. In summary, compared to CIoU, EIoU accelerates the convergence, increases the regression accuracy, and improves the training sample imbalance by adding focal loss.

## 4. Comparative Analysis Following the Algorithm Improvements

### 4.1. Model Evaluation Indicators

Accuracy and recall serve as two important metrics that can evaluate the object detection system performance of yolov5. Accuracy refers to the amount of correctly detected objects by the object detection system, while recall refers to the probability that a real object is detected [34]. In general, a higher correctness and recall indicate a better system performance. In this paper, the mean average precision (mAP), precision (P), and recall (R) are used as the evaluation indexes of the model performance with the following formulas:

(7) $$P=\frac{\mathrm{TP}}{\mathrm{TP}+\mathrm{FP}}\;,$$

(8) $$R=\frac{\mathrm{TP}}{\mathrm{TP}+\mathrm{FN}},$$

where TP is the number of positive samples predicted by positive samples; FP is the number of positive samples predicted by negative samples; FN is the number of negative samples predicted by positive samples. Furthermore, the mAP refers to the average of all AP types in all images.

### 4.2. Ablation Experiments

To verify the effectiveness of the improved yolov5 algorithm, we designed the ablation experiment. Table 2 reports the test results.

It can be seen from Table 3 that the model is improved by adding the lightweight upsampling operator CARAFE and the improved loss function. The model performance obtained by adding the lightweight upsampling operator CARAFE and improving the loss function is the best. The mAP_0.5 value of the model reaches 95.4%, which is 3.2% higher than that of the original yolov5 s. The average detection time was also reduced by 11 ms, and the model size was reduced by 2.21 MB.

### 4.3. Comparison of Results Before and After the Improvements

To better verify the improvement in the improved yolov5 algorithm and the unimproved yolov5 algorithm, the same tomato seedling stem data set is detected under the two algorithms before and after the improvement. Under the same number of iterations, the performance metrics (recall, precision, and mAP_0.5) of the improved and original yolov5 algorithms were compared (Table 3).

**Figure 10:**
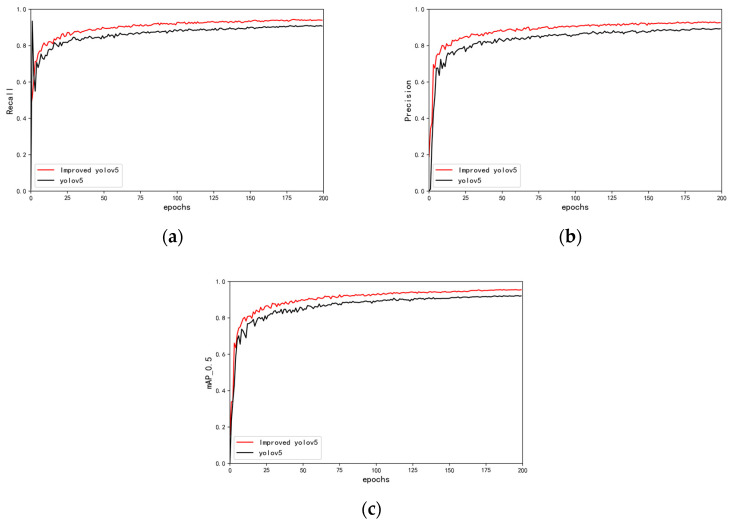
Comparison of indicators. (**a**) Recall; (**b**) precision; (**c**) mAP_0.5.

Table 4 reveals that our proposed algorithm improves on the original yolov5 algorithm. In particular, our improved algorithm achieves a recall of 94.1%, precision of 92.6%, and mAP_0.5 of 95.4%, which exceed the corresponding values for the original algorithm by 3.3%, 3.3%, and 3.2%, respectively. Figure 10 depicts the recall, precision, and mAP_0.5 of the improved and original algorithms.

**Figure 11:**
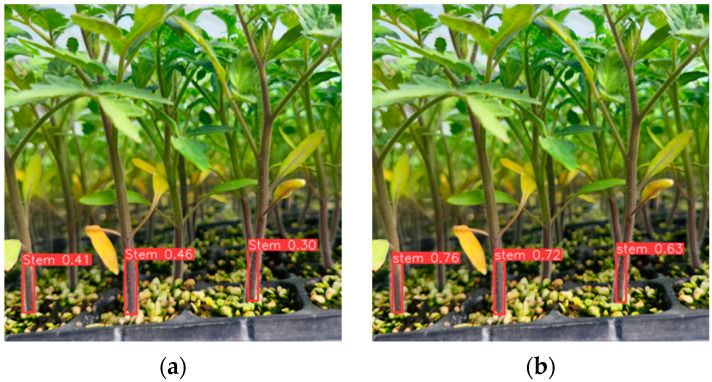
Test results before and after using the improved algorithm. (**a**) Raw test results; (**b**) Improved test results.

Figure 11 reveals that the detection results of the proposed algorithm are better compared to those of the original algorithm. The improved detection results have a higher confidence level, indicating that the identification of tomato seedling stems is more accurate. The improved algorithm improves the problem of low detection accuracy for tomato seedling stem recognition, thus enhancing the detection effect.

### 4.4. Performance Comparison with Other Detection Algorithms

To evaluate the superiority of our proposed algorithm in the detection of tomato seedling stems, we compare it with other typical target detection algorithms, namely yolov5, yolov8, and Faster R-CNN. The data set in the previous section was used for the comparison under the same conditions. Table 4 reports the results.

**Figure 12:**
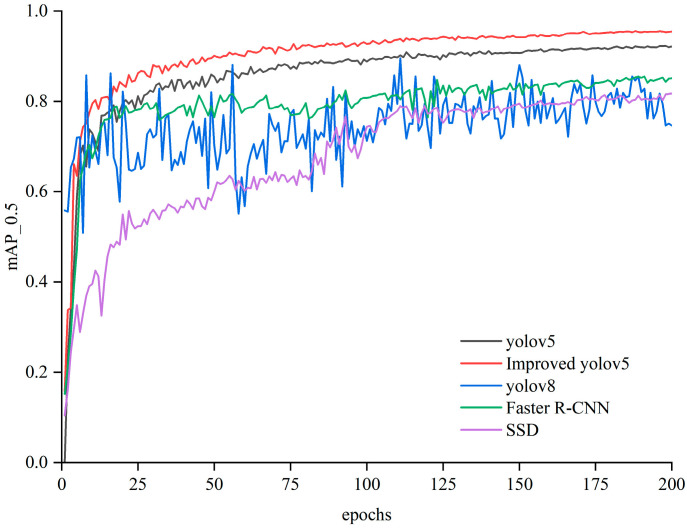
Performance comparison of different target detection algorithms.

The mAP_0.5 of the improved algorithm proposed in this paper reaches 95.4%, which is 3.2%, 12.9%, and 10.6% higher than that of yolov5, yolov8, and Faster R-CNN, respectively. Therefore, the improved algorithm performs better than the current mainstream target detection algorithms, with a marked improvement in the mAP_0.5. Figure 12 compares the mAP_0.5 values of the four detection algorithms.

## 5. Validation Tests

### 5.1. Test Scheme

**Figure 13:**
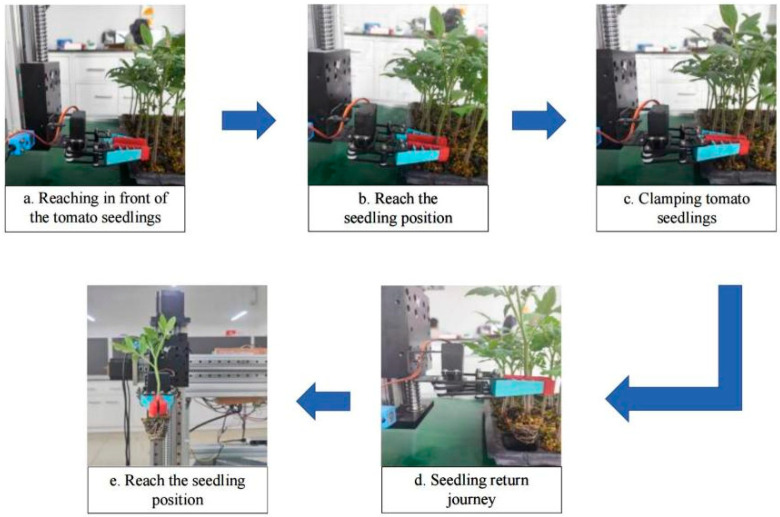
Seedling extraction process.

Seedling extraction testing of tomato hole tray seedlings was performed on the constructed test platform. The seedling extraction test begins by placing the hole tray seedling on the seedling extraction device. The camera then obtains the stem information of the tomato seedling, controls the mechanical claw to grasp the tomato seedling, and observes the seedling extraction situation of the device. According to our morphological analysis of the tomato plug seedlings, the seedling age of the tomato plug seedlings in this experiment was 40 days. The tomato plug seedlings were taken by the device, observed, and the relevant data were recorded. Figure 13 presents the seedling-picking process of the seedling-picking device.

### 5.2. Seedling Test Evaluation Index

We evaluated the performance of the constructed platform based on the following indicators:

(1) Picked seedling success rate: The tomato seedlings were removed from the plug and sent to the designated position. The completion of the process was taken to denote the successful picking of the seedlings. The success rate was determined as follows:
  (9) $$\alpha =\frac{n}{N}\;\times \;100\%,$$
  where α is the success rate of the seedling extraction, %; n is the number of tomato seedlings successfully extracted; and N is the total number of tomato seedlings.
(2) Tomato seedling damage rate: during the seedling extraction process, seedlings may be damaged by the grasping of the mechanical claw. This can affect the subsequent planting. Thus, the tomato seedling damage rate is used as a test evaluation index. Here, we define the damage to the tomato seedling as the rupture of the tomato seedling stalks and leaves. The damage rate is calculated as follows:
  (10) $$\beta =\frac{m}{M}\;\times \;100\%,$$
  where β is the damage rate of tomato seedlings, %; m is the number of tomato seedlings damaged in the process of grasping the seedlings, and M is the total number of tomato seedlings.

### 5.3. Seedling Test Results

Ten tomato plug seedling-picking experiments were performed. The seedling-picking experiment used 6 × 12 tomato plug seedlings as the test objects. Table 5 reports the test results.

The damage rate, success rate, and average success rate were determined as 7.2%, 90–96%, and 92.9%, respectively. Our analysis reveals that the main factor affecting the success rate of the seedling pick-up is the recognition of the tomato seedling stems. When the system does not identify tomato seedlings with poor growth status, the mechanical claw does not accept the command to remove the seedlings. At this time, the seedling pick-up success rate will not be affected by the inability to grasp seedlings with poor growth status. Therefore, further in-depth research on the stem identification of tomato seedlings is required.

## 6. Discussion and Conclusions

### 6.1. Discussion

Tomato transplanting is a key component of tomato production, and improving tomato transplanting efficiency and quality is currently an important topic. Numerous scholars have designed transplanting devices to reduce labor input and improve transplanting efficiency. Ren et al. [3] proposed a telescopic tomato seedling extraction device that realizes seedling extraction by clamping the substrate with minimal damage to the substrate. Jin et al. [35] designed an automatic tomato transplanter that also employs a grasping substrate for seedling picking, yet it lacks efficiency. To overcome the problem of damage to the substrate during the transplanting process, Liu et al. [36] employed the mechanical properties of tomato seedling substrate transplanting machine actuators for parameter optimization to provide a reference for subsequent research. It can be seen that the transplanting device designed by most scholars at this stage is to clamp the matrix to realize the seedling-picking operation, which does not apply to the cultivation mode of the sand matrix in southern Xinjiang. Based on this, we designed a stem-clamping seedling-picking device coupled with a camera. The proposed device can achieve automatic seedling picking with satisfactory results, with a damage rate of 7.2% and an average seedling picking success rate of 92.9%. This study is of great significance to the development of facility agricultural mechanization in southern Xinjiang.

At present, the designed device needs to be further optimized. Through analysis, we found that the claw of the seedling-picking device will damage individual fragile tomato seedlings during the picking process, failing the seedling-picking operation. In future work, the design of mechanical claws should be the focus of research to facilitate the use of different crop transplanting operations. In addition, at this stage, the device is in the laboratory stage and has not been applied in the field. In the field application, it may be affected by environmental interference, which will affect the detection performance. In the follow-up work, we will further study the identification of stems.

### 6.2. Conclusions

To evolve beyond the manual transplanting of tomato plug seedlings, this paper designs a stem-type tomato seedling transplanting and picking device based on machine vision. The key results and conclusions are summarized as follows:

(1) According to the basic characteristics of the tomato seedlings, the suitable transplanting period for tomato seedlings is determined as 40 days. Moreover, the depth information of the tomato stem grabbing point is obtained by integrating camera depth information into the proposed approach.
(2) An improved yolov5 algorithm is proposed to identify the tomato seedling stems. The lightweight upsampling operator CARAFE is added to the original yolov5 algorithm, and the loss function is improved. The improved algorithm is improved compared with the original yolov5 algorithm. The recall rate of the improved algorithm reaches 94.1%, the accuracy rate reaches 92.6%, and mAP_0.5 reaches 95.4%.
(3) A seedling-picking device is designed, and seedling-picking verification tests are conducted on the designed device, indicating that the overall operation requirements are met. The seedling damage rate of the device is 7.2%, the success rate of seedling-picking is between 90–96%, and the average success rate is 92.9%.

The purpose of this paper is to reduce the labor costs in facility transplanting and to realize intelligent transplanting. Future research will focus on designing seedling claws that can adapt to different crops and reduce seedling damage.

## Figures and Tables

**Table 1:** Morphological parameters of tomato hole tray seedlings.

Parameter	30 Days	35 Days	40 Days	45 Days	50 Days
Average plant height/cm	10.81	13.62	15.81	16.18	16.71
Plant height variance/cm	0.55	0.79	0.99	0.88	0.83
Average stem thickness/mm	2.75	3.03	3.33	3.35	3.38
Stem thickness variance/mm	0.04	0.04	0.06	0.04	0.04

**Table 2:** Ablation test results. ‘√’ indicates an improvement in the module, while ‘×’ indicates no improvement in the module.

Algorithm	CARAFE	EIOU	mAP_0.5/%	Average Test Time/ms	Model Size/MB
yolov5	×	×	92.2	47	6.35
yolov5 + EIOU	×	√	93.2	43	4.93
yolov5 + CARAFE	√	×	93.6	38	4.21
Proposed algorithm	√	√	95.4	36	4.14

**Table 3:** Comparison of performance indicators.

Algorithm	Recall/%	Precision/%	mAP_0.5/%
yolov5	90.8	89.3	92.2
Improved yolov5	94.1	92.6	95.4

**Table 4:** Comparison of different target detection algorithms.

Algorithm	mAP_0.5/%	Backbone
yolov5	92.2	CSPDarknet53
Improved yolov5	95.4	CSPDarknet53
yolov8	82.5	Darknet53
Faster R-CNN	84.8	ResNet-50

**Table 5:** Planting results of the device.

Test Times	Success Rate/%	Damage Rate/%
1	94.4	8.8
2	90.3	6.1
3	93.1	7.4
4	94.4	7.3
5	90.3	9.2
6	95.8	5.8
7	94.4	5.9
8	91.7	7.6
9	93.1	6.0
10	91.7	7.6
Average	92.9	7.2

## Data Availability

The data presented in this study are available on request from the corresponding author.
